# Vascular NRP2 triggers PNET angiogenesis by activating the SSH1-cofilin axis

**DOI:** 10.1186/s13578-020-00472-6

**Published:** 2020-09-23

**Authors:** Xi Luo, Jiang-yi He, Jie Xu, Shao-yi Hu, Bang-hui Mo, Qiu-xia Shu, Can Chen, Yu-zhu Gong, Xiao-long Zhao, Gan-feng Xie, Song-tao Yu

**Affiliations:** 1 Department of Oncology, Southwest Hospital, Third Military Medical University (Army Medical University), Chongqing, People’s Republic of China; 2 Department of Urology, The Second Affiliated Hospital, Third Military Medical University (Army Medical University), Chongqing, People’s Republic of China; 3 Nursing Division, Southwest Hospital, Third Military Medical University (Army Medical University), Chongqing, People’s Republic of China; 4 Department of Thoracic Surgery, Institute of Surgery Research, Daping Hospital, Third Military Medical University (Army Medical University), Chongqing, People’s Republic of China

**Keywords:** Neuropilin 2, Angiogenesis, SSH1, Cofilin, Pancreatic neuroendocrine tumor

## Abstract

**Background:**

Angiogenesis is a critical step in the growth of pancreatic neuroendocrine tumors (PNETs) and may be a selective target for PNET therapy. However, PNETs are robustly resistant to current anti-angiogenic therapies that primarily target the VEGFR pathway. Thus, the mechanism of PNET angiogenesis urgently needs to be clarified.

**Methods:**

Dataset analysis was used to identify angiogenesis-related genes in PNETs. Immunohistochemistry was performed to determine the relationship among Neuropilin 2 (NRP2), VEGFR2 and CD31. Cell proliferation, wound-healing and tube formation assays were performed to clarify the function of NRP2 in angiogenesis. The mechanism involved in NRP2-induced angiogenesis was detected by constructing plasmids with mutant variants and performing Western blot, and immunofluorescence assays. A mouse model was used to evaluate the effect of the NRP2 antibody in vivo, and clinical data were collected from patient records to verify the association between NRP2 and patient prognosis.

**Results:**

NRP2, a VEGFR2 co-receptor, was positively correlated with vascularity but not with VEGFR2 in PNET tissues. NRP2 promoted the migration of human umbilical vein endothelial cells (HUVECs) cultured in the presence of conditioned medium PNET cells via a VEGF/VEGFR2-independent pathway. Moreover, NRP2 induced F-actin polymerization by activating the actin-binding protein cofilin. Cofilin phosphatase slingshot-1 (SSH1) was highly expressed in NRP2-activating cofilin, and silencing SSH1 ameliorated NRP2-activated HUVEC migration and F-actin polymerization. Furthermore, blocking NRP2 in vivo suppressed PNET angiogenesis and tumor growth. Finally, elevated NRP2 expression was associated with poor prognosis in PNET patients.

**Conclusion:**

Vascular NRP2 promotes PNET angiogenesis by activating the SSH1/cofilin/actin axis. Our findings demonstrate that NRP2 is an important regulator of angiogenesis and a potential therapeutic target of anti-angiogenesis therapy for PNET.

## Background

Pancreatic neuroendocrine tumors (PNETs) are a rare cancer with an incidence of less than 1 per 100,000 persons per year and represent 1–2% of all pancreatic neoplasms [1]. In recent years, the incidence of PNETs has increased due to improvements in the pathologic and diagnostic techniques used to identify these tumors [2]. Surgical resection provides a potential cure, although 85% of PNET cases are unresectable at the time of diagnosis [3]. Medical oncologic intervention is recommended by PNET guidelines (European Society for Medical Oncology, North American Neuroendocrine Tumor Society) to improve survival in individuals with advanced disease, but PNETs show high resistance to routine chemotherapy. Due to this phenomenon, it is necessary to identify other therapeutic interventions for PNETs. PNETs are highly vascularized neoplasms; therefore, pharmaceutical treatments against angiogenesis are an interesting therapeutic option for advanced cases. Unfortunately, clinically available anti-angiogenic drugs targeting the VEGF/VEGFR pathway failed to improve PNET survival rates [4]. Recent studies have found that tumor angiogenesis is not completely dependent on the VEGF/VEGFR pathway [5]. Thus, to develop therapeutic agents for PNETs, it is essential to explore additional anti-angiogenic targets.

Neuropilin 2 (NRP2), a cell surface transmembrane protein, was originally characterized as a receptor for type 3 semaphorins (such as SEMA3) and as a co-receptor of VEGFR2 [6]. NRP2 is primarily expressed in the nervous and vascular systems and is important for the development of embryos by enhancing VEGF-VEGFR binding [6]. Recent studies have reported that the neuropilin family is involved in tumor metastasis and is correlated with poor prognosis [7, 8]. Moreover, NRP2 has been found to be highly expressed in pancreatic islet cells and endocrine pancreatic tumors [9]. However, the function and mechanism of NRP2 in PNET angiogenesis is unknown.

Angiogenesis is an actin-dependent process, which is demonstrated by its sensitivity to actin polymerization [10, 11]. The rate of conversion of monomeric globular actin (G-actin) to filamentous actin (F-actin) contributes to actin polymerization. Cofilins are actin-binding proteins that play an essential role in regulating actin filament dynamics and reorganizing actin structures by stimulating the breakdown and depolymerization of actin filaments [12]. Previous studies showed that inhibiting cofilin expression decreased human umbilical vein endothelial cells (HUVEC) migration and angiogenesis [13]. Cofilin activity is regulated by phosphorylation at the serine residue at position 3 (Ser-3): phosphorylation of this residue inactivates the protein and is mediated by LIM kinases (LIMKs) and testicular protein kinases (TESKs) [14–16], whereas dephosphorylation of Ser-3 activates the protein and is mediated by slingshot protein phosphatases (SSHs) [17] and the haloacid dehalogenase chronophin (CIN) [18]. Furthermore, SSH1 has been reported to accumulate in membrane protrusions and mediate actin remodelling in HUVECs [19]. However, the role of the SSH1-cofilin pathway in PNET angiogenesis is still unknown.

Here, we show that tumor angiogenesis triggered by vascular NRP2 is driven by the promotion of F-actin polymerization in HUVECs. We further demonstrate that (1) high levels of NRP2 expression positively correlate with PNET vascularity; (2) NRP2 modulates angiogenesis by promoting HUVEC migration via a VEGF/VEGFR2-independent pathway; (3) NRP2 induces F-actin polymerization by activating the actin-binding protein cofilin; (4) NRP2 upregulates cofilin activity by promoting SSH1 expression; and (5) inhibition of NRP2 suppresses PNET angiogenesis and tumor growth in vivo. High expression of NRP2 was associated with poor prognosis in PNET patients. Therefore, vascular NRP2 triggers PNET angiogenesis via activation of the SSH1-cofilin pathway.

## Results

### NRP2 expression is positively correlated with PNET vascularity

**Fig. 1:**
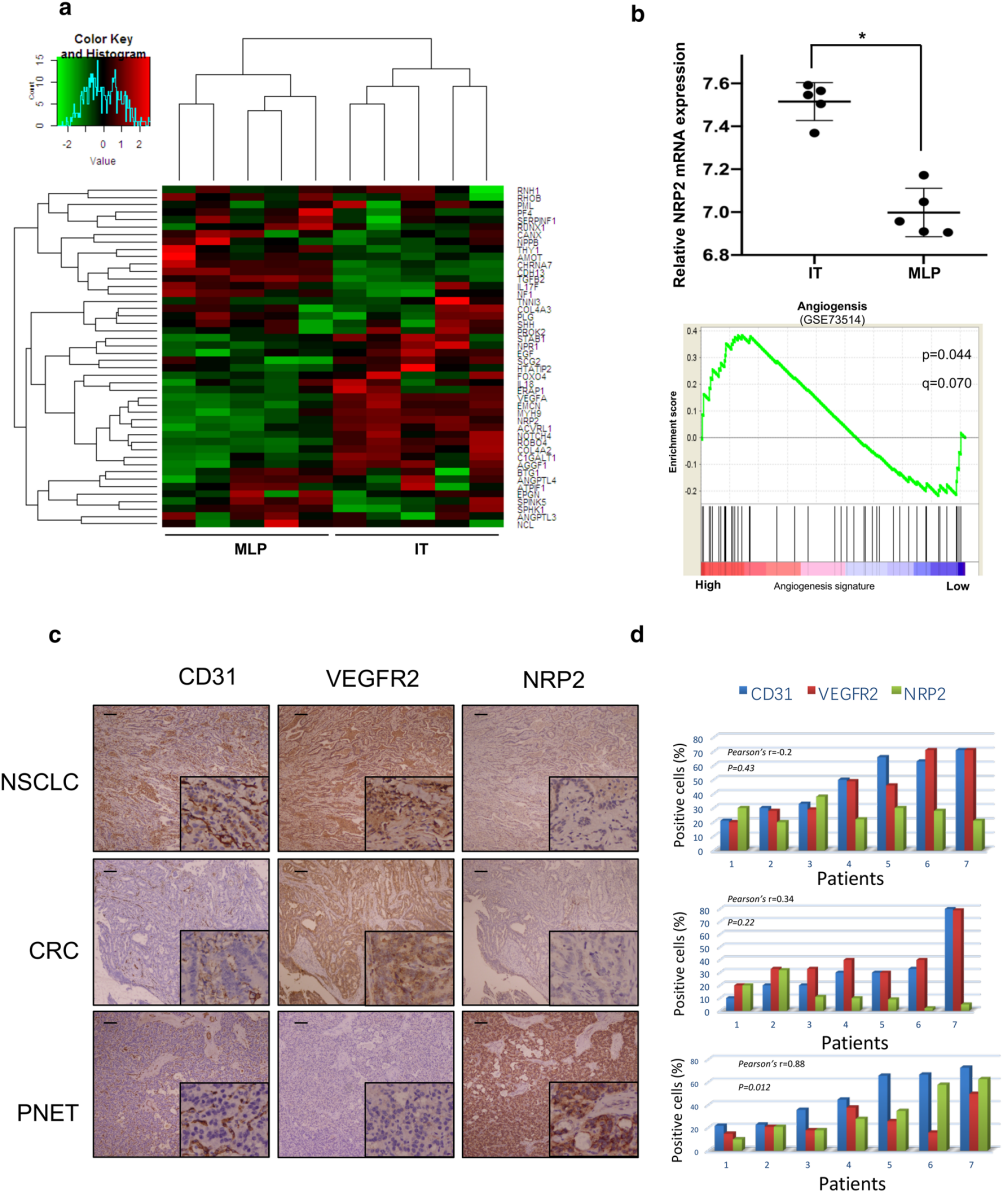
NRP2 expression is positively correlated with PNET vascularity. **a** Heatmap summarizing the angiogenesis process signature (GSE73514) in metastatic-like primary (MLP) tumor and islet tumor (IT) samples. **b** *Top:* Comparison of NRP2 mRNA expression between the MLP and IT groups. *Bottom:* GSEA mountain plot showing a strong association between the MLP and IT groups. The data are presented as the means ± SD. *P ≤ 0.05 by Student’s t test. C, Representative expression of CD31, VEGFR2 and NRP2 in non-small-cell lung cancer (NSCLC), colorectal cancer (CRC) and pancreatic neuroendocrine tumor (PNET) specimens according to immunohistochemistry assays. **d** Correlation histograms of CD31, VEGFR2 and NRP2 expression in NSCLC, CRC and PNET specimens according to immunohistochemistry. Each number on the horizontal axis represents one specimen from the patients. The bars in the histograms show the mean percentage of positively staining cells under 5 randomly selected microscopic fields (20x). The Spearman R value and *P* value in the figures reflect the correlations of NRP2 and CD31 expression

Angiogenesis is a critical process in the growth and dissemination of PNETs, which suggests that anti-angiogenesis treatments may be a selective therapeutic option. The underlying mechanism driving angiogenesis in PNETs, however, is unclear. To better understand PNET angiogenesis, we examined the PNET dataset GSE73514, which includes 5 pairs of RIP1-TAG2 mouse models, and compared the transcription levels of genes associated with angiogenesis [23]. In the dataset, metastatic-like primary (MLP) tumors were comparatively less vascularized than islet tumors (ITs). Unexpectedly, VEGFR and VEGF were not related to the vascular density in PNET tissues (Fig. 1a). Furthermore, transcript levels of NRP2, a VEGFR2 co-receptor, were significantly higher in IT samples than in MLP tumor samples (Fig. 1b), which suggests that NRP2, not VEGFR2, is associated with PNET angiogenesis. To verify this hypothesis, an immunohistochemical staining assay was performed on 7 PNET tissues, with 7 colorectal cancer (CRC) and 7 non-small-cell lung cancer (NSCLC) specimens as positive controls owing to their sensitivity to VEGFR-related anti-angiogenesis effects. As Fig. 1c shows, VEGFR2 expression positively correlated with the angiogenesis marker CD31 in 7 CRC specimens and 7 lung cancer specimens. Moreover, CD31 expression was positively correlated with NRP2 but not with VEGFR2 in PNET specimens (*p* = 0.012) (Fig. 1d), but this was not the case in NSCLC and CRC tissues. Therefore, NRP2 expression is positively correlated with PNET vascularity.

### NRP2 modulates angiogenesis by promoting HUVEC migration via a VEGF/VEGFR2-independent pathway

**Fig. 2:**
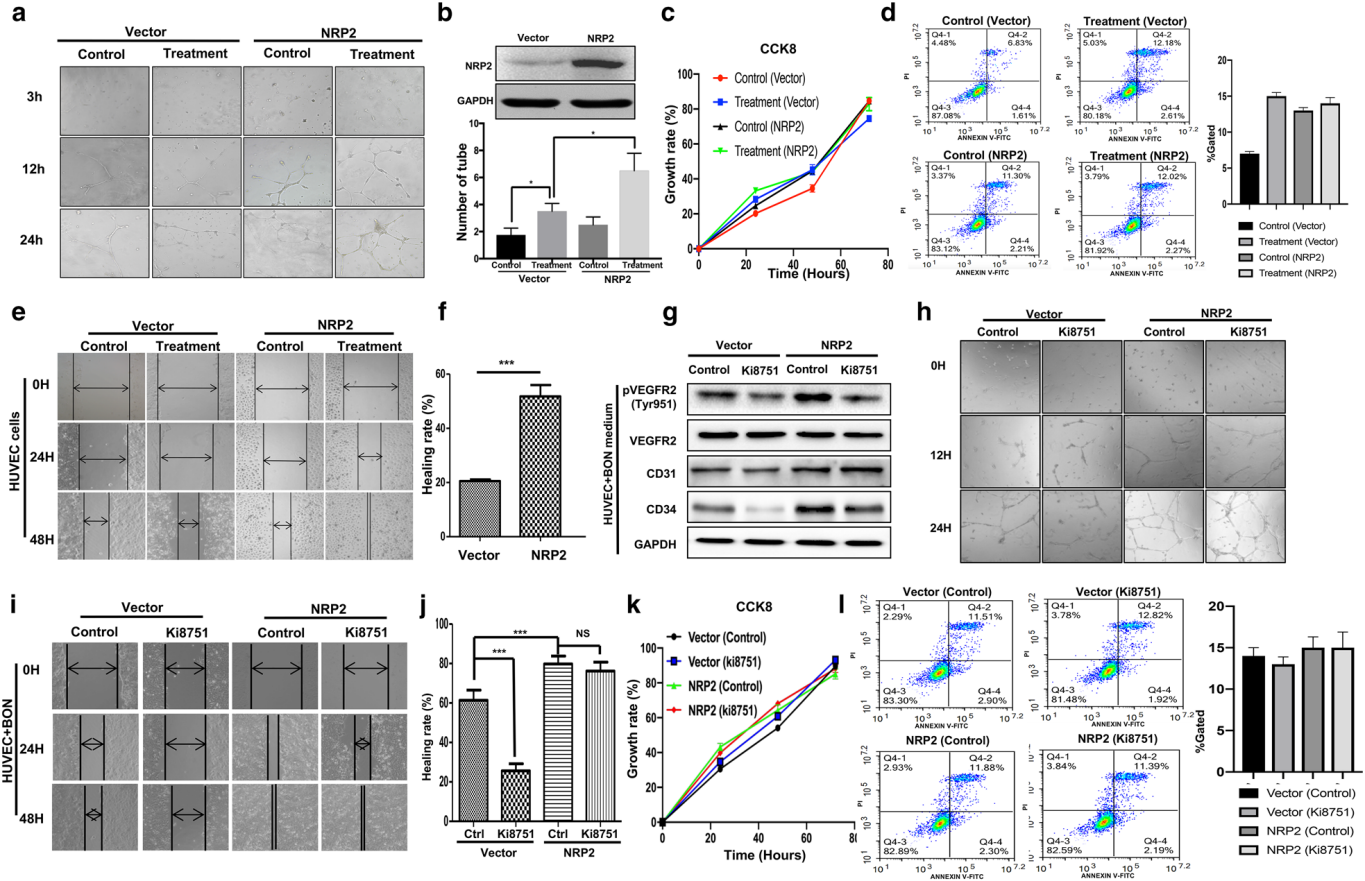
NRP2 modulates angiogenesis by promoting HUVEC migration via a VEGF/VEGFR2-independent pathway. **a** HUVECs were cultured in the presence or absence of conditioned medium from BON cells (treatment and control, respectively) and then transfected with a vector control or NRP2 overexpression plasmid before they were seeded for the capillary tube formation assay. Representative images at 4, 12 and 24 h after plating are shown. **b** Quantification of the number of complete and broken tubes at 6 h from a representative experiment. Data are shown as the mean ± SD of three independent experiments. *P ≤ 0.05 by Student’s *t* test. **c** HUVECs were treated with conditioned medium from BON cells for 24 h and then transfected with a vector or NRP2 overexpression plasmid before they were subjected to a CCK8 assay. **d** After HUVECs were cultured in the presence or absence of conditioned medium from BON cells (treatment and control, respectively) and transduced with the NRP2-overexpressing plasmid, flow cytometry was performed to assess apoptosis. **e** Representative images for the wound-healing assay at 0, 24 and 48 h after scratching for the 4 different cell groups (HUVECs with or without NRP2 overexpression cultured in the presence or absence of conditioned medium from BON cells). **f** Quantification of the healing rate at 48 h after wound-healing assays in HUVECs cultured in the presence or absence of conditioned medium from BON cells followed by transfection with empty vector or NRP2 plasmid. The data are shown as the means ± SD of three independent experiments. ***P ≤ 0.001 by Student’s *t* test. **g** HUVECs were transfected with empty vector or an NRP2 overexpression plasmid and then treated with the VEGFR2-specific inhibitor KI8751. Western blotting assays were performed to determine the levels of VEGFR2 phosphorylation at Tyr951 as well as the total protein levels of VEGFR2, CD31, CD34 and GAPDH. **h** Control and NRP2-overexpressing HUVECs were treated with KI8751 and PBS and evaluated for tube formation. **i** After HUVECs were cultured in the presence or absence of conditioned medium from BON cells for 24 h, they were transfected with a vector or NRP2 overexpression plasmid. These cells were subsequently treated with PBS (control) or KI8751, and a wound-healing assay was performed. Representative image of three independent experiments is shown. **j** Qualification of the wound-healing rate at 48 h in HUVEC-vector or HUVEC-NRP2 cells treated with PBS or KI8751. The data are shown as the mean ± SD of three independent experiments. **P ≤ 0.01 by Student’s *t* test. **k** After HUVECs were transfected with empty vector or an NRP2 overexpression plasmid, they were treated with PBS or KI8751 and subjected to the CCK8 assay at 24, 48 and 72 h after treatment. **l** Flow cytometry assay was performed in the 4 cell groups described in Fig. 2k

To elucidate whether NRP2 expression promotes PNET angiogenesis, we first ectopically expressed NRP2 in HUVECs. We treated HUVECs with conditioned medium from pancreatic tumor cells (BON cells) to mimic the environment in which vascular epithelial cells grow in PNETs. We then compared the tube formation ability of parental HUVECs and BON medium-treated HUVECs in Matrigel. After HUVECs were cultured overnight in conditioned medium from BON cells, the data show that ectopic NRP2 expression in HUVECs significantly extended capillary tube length and decreased the abundance of broken capillary tubes (Fig. 2a, b). The same results were shown in another PNET cell line, beta-TC3 (Additional file 1. Figure S1a). To determine how NRP2 promotes tube formation, we performed CCK-8, flow cytometry and wound-healing assays to assess its effect on cell proliferation and migration. Although overexpression of NRP2 failed to affect the proliferation and apoptosis of HUVECs (Fig. 2c, d), it dramatically promoted the migration of HUVECs cultured in the presence of conditioned medium from BON cells (Fig. 2d). The healing rate of HUVECs overexpressing NRP2 reached 55%, which was significantly higher than the 20% healing rate observed in the control group (*p* < 0.001) (Fig. 2e). Collectively, these data suggest that NRP2 induces angiogenesis by promoting HUVEC migration when cultured ins conditioned medium from PNET cell but not by promoting cell proliferation.

NRP2 is a co-receptor of VEGFR2 that promotes angiogenesis during embryonic development [24]. Recent studies have suggested that NRP2 activates downstream pathways independent of VEGFR2 [25]. Furthermore, we found that NRP2 expression, but not VEGFR2 expression, was positively correlated with CD31 expression in PNET specimens (Fig. 1c), which suggests that NRP2 promotes HUVEC migration in a VEGF/VEGFR2-independent manner. Herein, we demonstrated that overexpression of NRP2 in HUVECs, along with treatment with conditioned medium from BON cells, increased the protein levels of both CD31 and CD34 (Fig. 2f) and dramatically promoted HUVEC migration (Fig. 2g, h), neither of which could be significantly blocked by simultaneous inhibition of VEGFR2; these results are consistent with the data in Fig. 1d. In addition, inhibiting VEGFR2 activity did not decrease tube formation by NRP2 (Fig. 2h), and NRP2 overexpression and/or VEGFR2 inhibition failed to affect cell proliferation and apoptosis (Fig. 2i and l). Taken together, our data indicate that NRP2 modulates angiogenesis by promoting HUVEC migration in the presence of conditioned medium from pancreatic cells via a VEGF/VEGFR2-independent pathway.

To further clarify whether NRP2 promotes HUVEC migration in lung cancer and CRC models, HUVECs were transfected with an NRP2 plasmid and then treated with conditioned medium from BON, A549 and SW480 cells. Wound-healing assays indicated that the BON cell conditioned medium promoted HUVEC migration, whereas neither the A549 nor SW480 cell conditioned media could promote the migration of HUVECs overexpressing NRP2 (Additional file 2. Figure S2a). While the healing rate of the BON group was increased by more than 80% upon ectopic expression of NRP2, the healing rates of the A549 and SW480 groups were less than 20% (Additional file 2. Figure S2a and S2b). Therefore, NRP2 is not involved in angiogenesis in CRC or lung cancer, nor does it promote HUVEC migration. Furthermore, NRP2 promotes cell migration in the presence of conditioned medium from pancreatic cancer cells but not from colorectal or lung cancer cells.

### NRP2 polymerized F-actin by activating the actin-binding protein cofilin

**Fig. 3:**
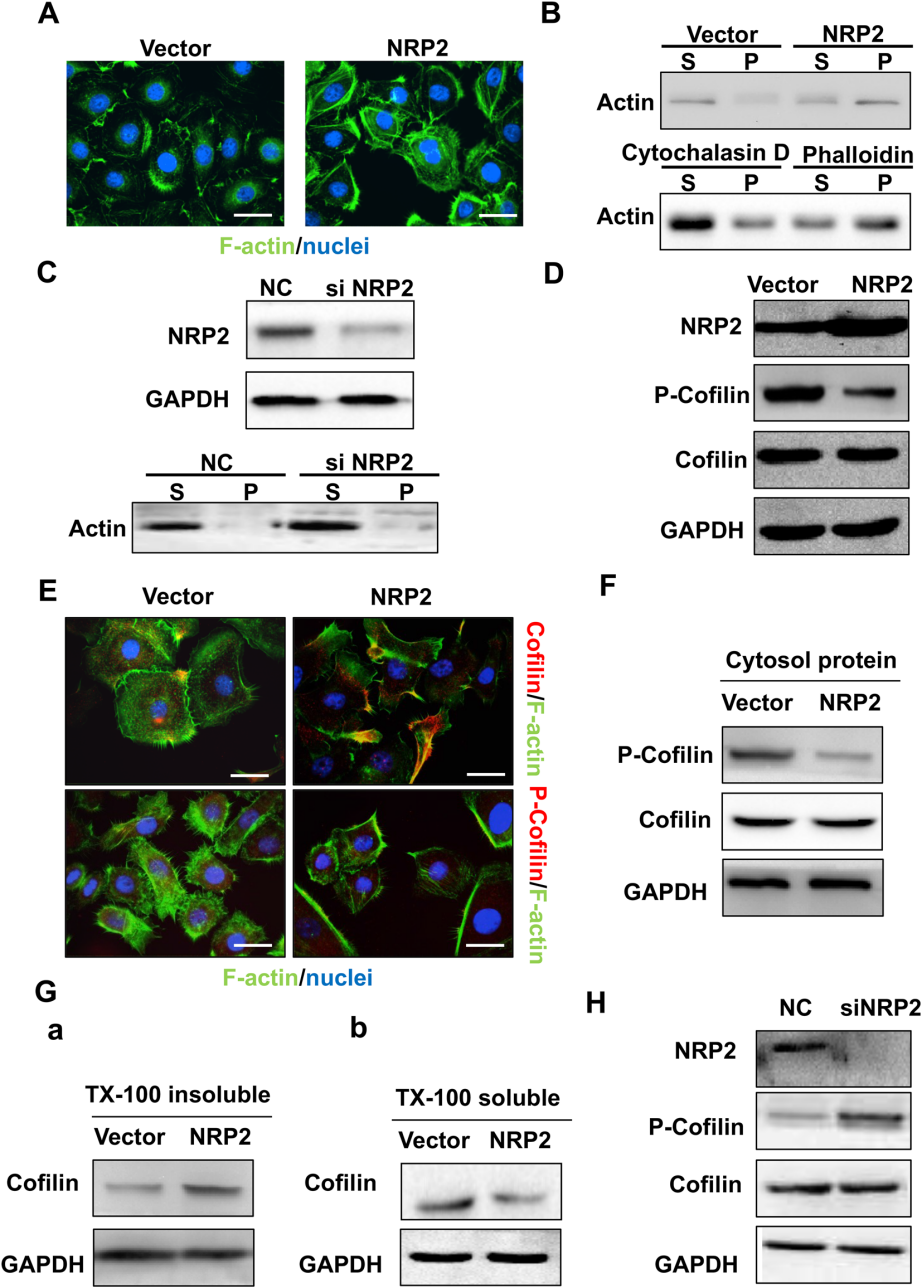
NRP2 induced F-actin polymerization via the active actin-binding protein cofilin. **a** Immunofluorescence analysis was performed using FITC-labelled phalloidin (F-actin; green), and the nuclei were stained with DAPI (blue). An overlay of the two fluorescent signals is shown (× 1000). **b** F-actin and G-actin fractions were prepared from HUVECs transfected with empty vector or NRP2 overexpression plasmid (Top). HUVECs were treated with either F-actin depolymerization factor (cytochalasin D) as a positive control or F-actin enhancing factor (phalloidin) as a negative control. The F-actin and G-actin fractions were prepared and subjected to Western blot analysis [S, supernatant fraction (G-actin); P, pellet fraction (F-actin)] (bottom). **c** HUVECs were transfected with si-control or si-NRP2 and subjected to Western blot analysis using the indicated antibodies. **d** After HUVECs were transfected with empty vector or an NRP2 overexpression plasmid, they were lysed and subjected to Western blot analysis with the indicated antibodies. **e** HUVEC-vector and HUVEC-NRP2 cells were coimmunostained with FITC-labelled F-actin and antibodies targeting total and phosphorylated cofilin. The fluorescent signals of cofilin or p-cofilin (red) along with F-actin (blue) are shown (× 1000). **f** HUVEC-vector and HUVEC-NRP2 cells were lysed using cytosol buffer and subjected to Western blotting with the indicated antibodies. **g** After HUVECs transfected with empty vector or an NRP2 overexpression plasmid were lysed in Triton X-100 buffer, the insoluble and soluble fractions were subjected to Western blotting. **h** After NRP2 was knocked down, HUVEC lysates were subjected to Western blot analysis with the indicated antibodies

Cell migration is physically mediated by the actin cytoskeleton and is initiated by the protrusion of the cell membrane. To determine the exact mechanism of NRP2-induced migration of HUVECs cultured with PNET conditioned medium, an actin cytoskeletal organization assay was performed. In vector cells treated with short-term conditioned media from BON cells, actin was diffusely distributed throughout the cytoplasm (Fig. 3a, left). By contrast, in NRP2-overexpressing cells, actin structures were indicated by larger actin-rich lamellipodia protrusions around the periphery of the cells with a few thin stress fibres located within the cell body (Fig. 3a, right). The rates of actin polymerization and depolymerization are important determinants of cell mobility, and a cellular F-actin/G-actin assay was carried out. Cells were transfected with NRP2, treated with the F-actin depolymerization factor cytochalasin D (negative control) or treated with the F-actin enhancing factor phalloidin (positive control). Our data showed that NRP2 overexpression strikingly increased the amount of F-actin similar to that observed in the phalloidin treatment group (P, Fig. 3b, right panel), whereas the F-actin depolymerization factor cytochalasin D failed to induce the formation of filaments (Fig. 3b, bottom left panel). Nevertheless, F-actin did not reorganize upon small interfering RNA (siRNA)-mediated silencing of NRP2 (Fig. 3c). Thus, NRP2-driven HUVEC migration is associated with reorganization of the actin cytoskeleton.

Cofilin, an actin-binding protein, regulates actin polymerization and depolymerization rates. Here, we found that NRP2 overexpression induced cofilin dephosphorylation at Ser 3 while the levels of total cofilin were unchanged (Fig. 3d). In NRP2-overexpressing cells, total cofilin colocalized with F-actin and was recruited to the leading edge (Fig. 3e, top), whereas the levels of phosphorylated cofilin were reduced in the cytoplasm (Fig. 3e, f). NRP2 overexpression induced cofilin enrichment in the detergent-insoluble fraction (Fig. 3G(a)). In control cells, cofilin was located mainly in the detergent-soluble fraction, but this decreased upon overexpression of NRP2 (Fig. 3G(b)). Moreover, silencing NRP2 induced cofilin phosphorylation at Ser 3 (Fig. 3h), which would lead to a reduction in cofilin activity. Thus, these data indicate that NRP2 induces cofilin activity to remodel actin fibres.

### Cofilin activity mediates NRP2-driven HUVEC migration and actin organization

**Fig. 4:**
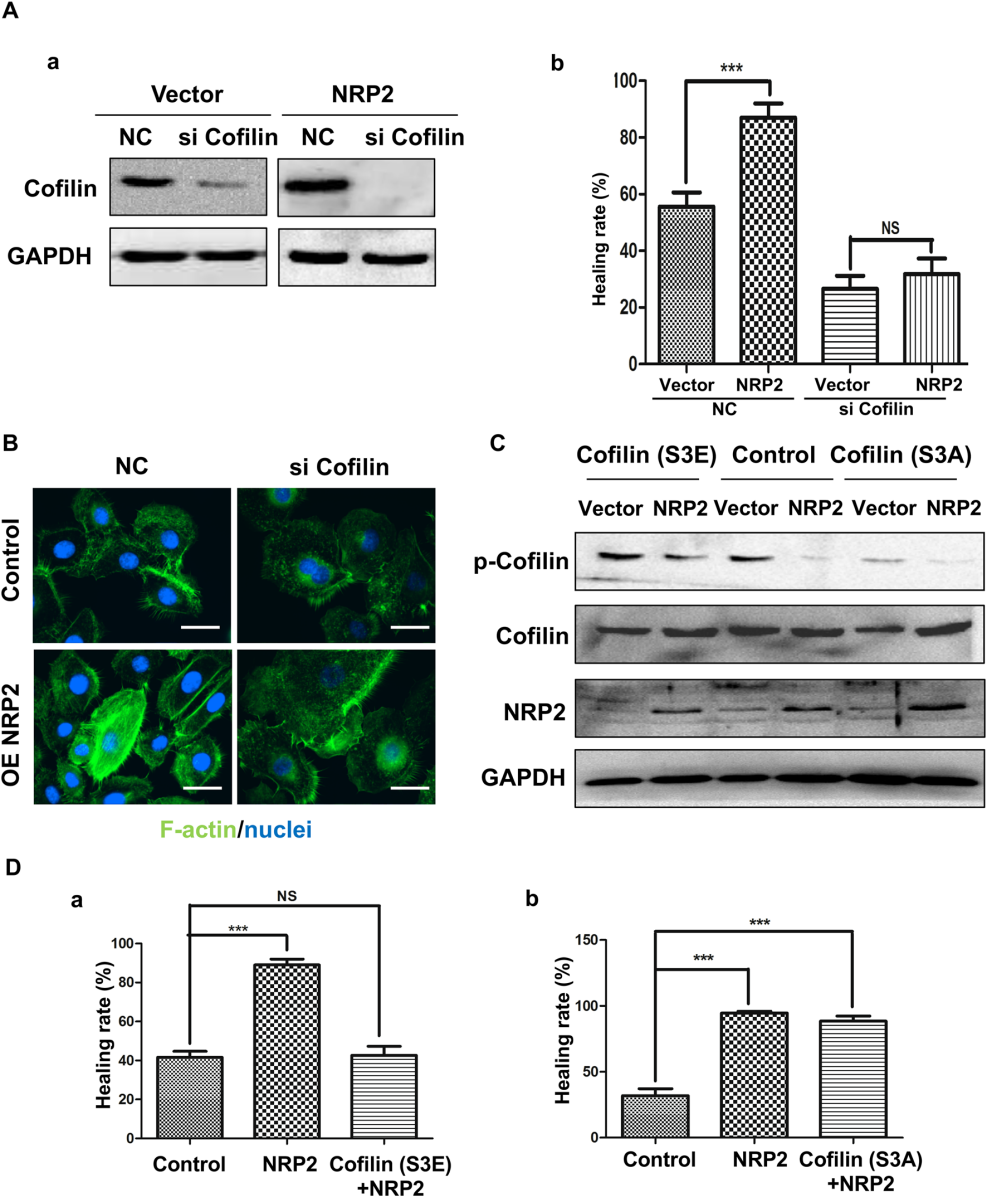
Cofilin activity mediates NRP2-driven HUVEC migration and actin organization. **A** HUVEC-vector and HUVEC-NRP2 cells were transfected with scramble or cofilin siRNA. **a** Cells were lysed and subjected to Western blot analysis with cofilin antibodies. **b** The migratory properties of the cells were analysed by the wound-healing assay (***P ≤ 0.001 by Student’s *t* test). The data are presented as averages from three independent experiments. **B** The fluorescent signals of F-actin (green) and nuclei (blue) are shown (× 1000). **C** HUVEC-vector and HUVEC-NRP2 cells were transfected with cofilin S3A or S3E before they were lysed and subjected to Western blot analysis with the indicated antibodies. **D** HUVEC-NRP2 cells were transfected with cofilin S3A (**a**) or cofilin S3E (**b**). The migratory properties of the cells were analysed by the wound-healing assay. The data are summarized from three independent experiments

To further address the dependence of NRP2-induced cell migration on cofilin, we downregulated endogenous cofilin using a specific siRNA, which caused an approximately 90% decrease in cofilin in both control and NRP2-overexpressing cells (Fig. 4A(a)). Our data then showed that NRP2 overexpression increased cell migration (Fig. 4A(b) and Additional file 3. Figure S3a) and induced actin reorganization (Fig. 4b), which could be abrogated by simultaneously knocking down cofilin. To further analyse whether cofilin activity contributed to NRP2-induced cell migration and actin polymerization, we expressed dominant-active or dominant-negative cofilin mutants in control and NRP2-overexpressing cells (Fig. 4c). Cofilin is inactivated by phosphorylation of Ser-3 near the N-terminus [26]. Expression of a cofilin S3E mutant, which mimics phosphorylation and inactivates cofilin, in NRP2-overexpressing cells failed to promote cell migration (Fig. 4D(a) and Additional file 3. Figure S3b), whereas expression of a cofilin S3A mutant, which is unable to be phosphorylated and thus activates cofilin, significantly promoted cell migration, mimicking the phenotype of NRP2 overexpression [Fig. 4D(b) and (Additional file 3. Figure S3c]. These results provide additional evidence that cofilin activity plays an essential role in NRP2-driven cell mobility.

### NRP2 upregulates cofilin activity by increasing SSH1 expression

**Fig. 5:**
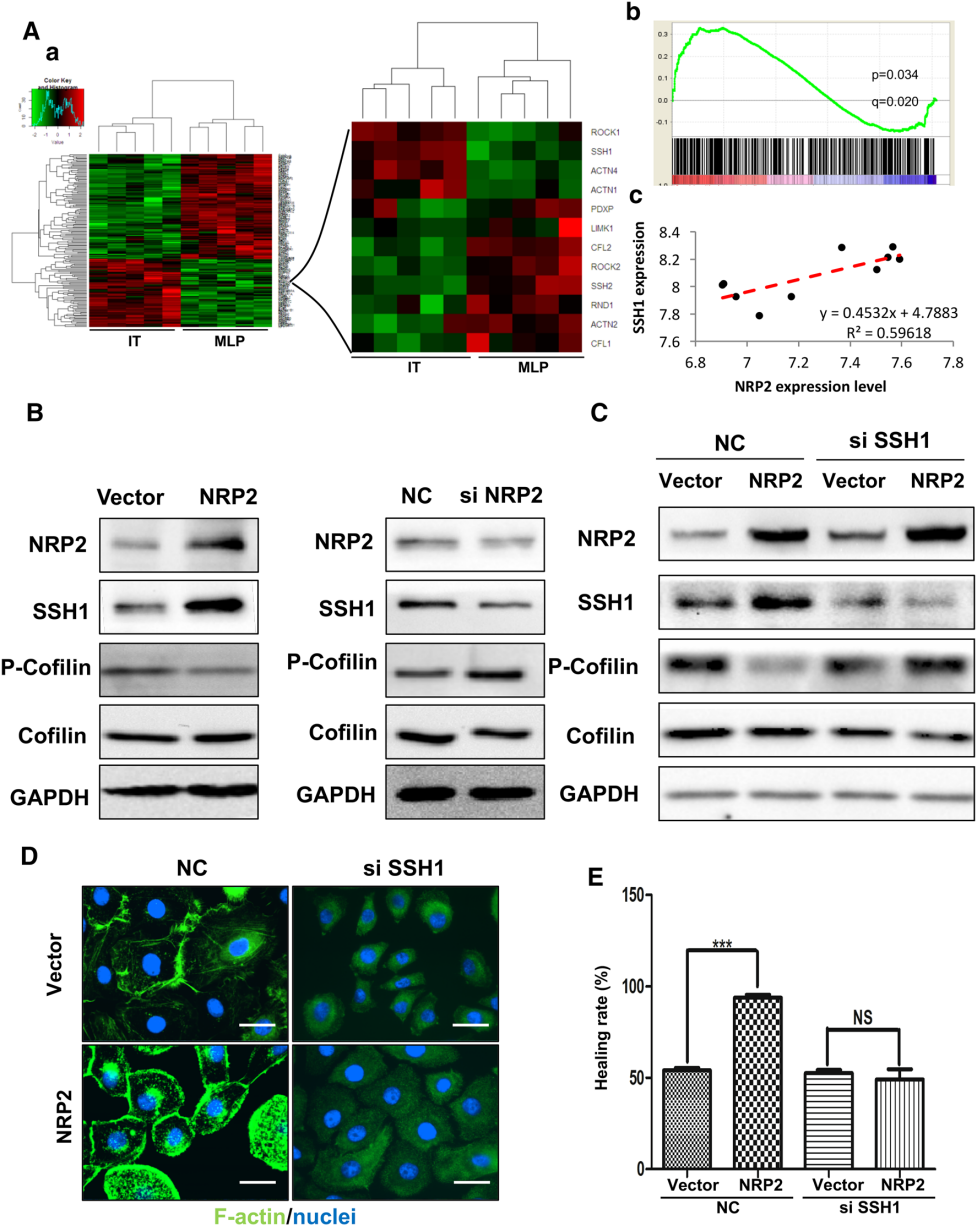
NRP2 upregulates cofilin activity by increasing SSH1 expression. **A** Heatmap of an NCBI dataset (GSE73514) of MLP tumors and ITs. Cluster analysis was performed according to an NRP2 transcription level difference of more than fivefold in 2 groups (red indicates high transcription levels, and blue indicates low transcription levels). **a** Cluster analysis results. **b** GSEA mountain plot showing a strong association between the MLP and IT groups. c, Correlation analysis of NRP2 and SSH1 transcription levels. **B** SSH1 expression in HUVEC-vector and HUVEC-NRP2 cells was detected by Western blot analysis (left panels). SSH1 expression in scramble siRNA- or NRP2 siRNA-treated cells was detected by Western blot analysis (right panels). **C** HUVEC-vector and HUVEC-NRP2 cells were treated with scramble or SSH1 siRNA. Cells were then lysed and subjected to Western blot analysis with the indicated antibodies. **D** The fluorescent signals of F-actin (green) and nuclei (blue) are shown (× 1000). **E** The migratory properties of the cells were analysed by the wound-healing assay. The data are presented as averages from 3 independent experiments (***P ≤ 0.001 by Student’s *t* test)

Phosphorylation of cofilin at Ser-3 near the N-terminus can be induced by several mechanisms. Thus, we conducted a pathway relationship analysis using the NCBI database (GSE73514) [23]. Enrichment analysis was carried out to identify potentially relevant pathways within which regulated genes were significantly enriched, and there were statistically significant differences in the observed GO pathways (Fig. 5A(a), left). The actin filament-based process (GO: 0030029) was significantly enriched in the IT group, which comprises richly vascularized tumor tissues and elevated NRP2 expression (Fig. 5A(a), right) (enrichment score = 0.33, P < 0.001, FDR q < 0.05) (Fig. 5A(b)). SSH1 expression was positively correlated with NRP2 expression (Fig. 5A(c)); in fact, SSH1 expression and transcription were increased in NRP2-overexpressing cells, while cofilin phosphorylation was decreased (Fig. 5b, left and Additional file 1. Figure S1b). After NRP2 was silenced, there was a decrease in SSH1 levels and an increase in cofilin phosphorylation (Fig. 5b, right). Silencing SSH1 blocked NRP2-induced cofilin dephosphorylation (Fig. 5c), and immunofluorescence assays showed that silencing SSH1 inhibited NRP2-induced F-actin polymerization (Fig. 5d) and HUVEC migration (Fig. 5e). Taken together, these data indicate that NRP2 inhibits cofilin phosphorylation by promoting the expression of SSH1.

### Downregulation of NRP2 suppresses PNET angiogenesis, slows tumor growth in vivo and extends patient survival

**Fig. 6:**
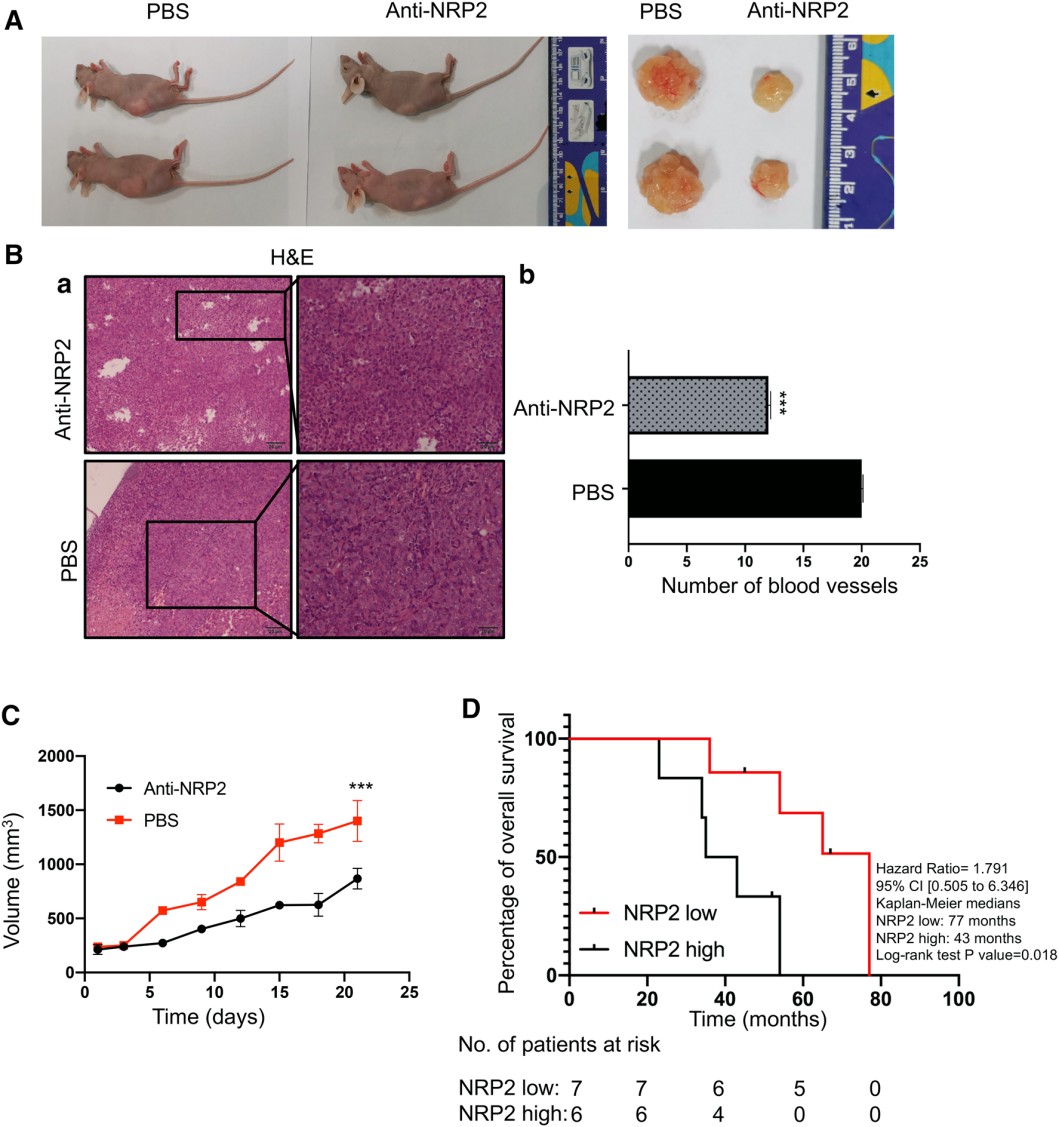
Downregulation of NRP2 suppresses PNET angiogenesis and tumor growth in vivo and correlates with increased survival in patients. **a** The xenograft experiments in vivo with the mouse model with BON cells. After mice were injected with anti-NRP2 antibody or PBS intraperitoneally, the xenografts were dissected. **b** H&E staining was performed to determine the number of vessels in the xenograft tumors. The vascular number was calculated as the mean counts of vessels in 5 fields under 20 × magnification. ***P ≤ 0.001 by Student’s *t* test. **c** Tumor sizes were measured every other day after injection with PBS or NRP2 antibody. **d** Survival curve of PNET patients in the high and low NRP2 expression groups

Lastly, we generated an orthotropic xenograft tumor model to identify the pro-angiogenic effect of NRP2 in vivo (Fig. 6a). After intraperitoneal injecting anti-NRP2 antibodies into xenograft tumors, visible angiogenesis was suppressed compared with that in mice injected with PBS (Fig. 6a). Moreover, the number of blood vessels was reduced by the presence of anti-NRP2 antibody (*p* = 0.0023 Fig. 6b). Tumor growth was significantly reduced in anti-NRP2-treated mice compared with PBS-treated mice (Fig. 6c). Moreover, NRP2 antibody failed to reduce the tumor sizes in CRC and lung cancer mouse models (Additional file 4. Figure S4). Thus, blocking NRP2 significantly reduced PNET angiogenesis in vivo. Next, 13 patients with PNET were recruited to verify the effect of NRP2 on survival (Table 1). Consistent with the mouse model findings, a survival analysis showed that the median survival was significantly longer in patients with low NRP2 expression than in those with high NRP2 expression (Fig. 6d). Taken together, these outcomes indicate that downregulation of NRP2 suppresses PNET angiogenesis and tumor growth in vivo and is associated with longer survival in humans.

**Table 1:** Characteristics of patients

NO	Gender	Age	Stages	Functional	WHO grade	NRP2 expression
1	Male	56	III	Yes	G2	Low
2	Male	54	IV	No	G1	Low
3	Male	67	II	No	G2	Low
4	Male	70	IV	Yes	G2	Low
5	Male	65	III	No	G1	Low
6	Female	61	IV	No	G2	Low
7	Female	59	II	No	G2	Low
8	Male	66	IV	Yes	G2	High
9	Male	68	IV	Yes	G2	High
10	Female	61	II	No	G1	High
11	Female	54	IV	No	G2	High
12	Female	58	IV	No	G2	High
13	Female	66	IV	No	G2	High

## Discussion

**Fig. 7:**
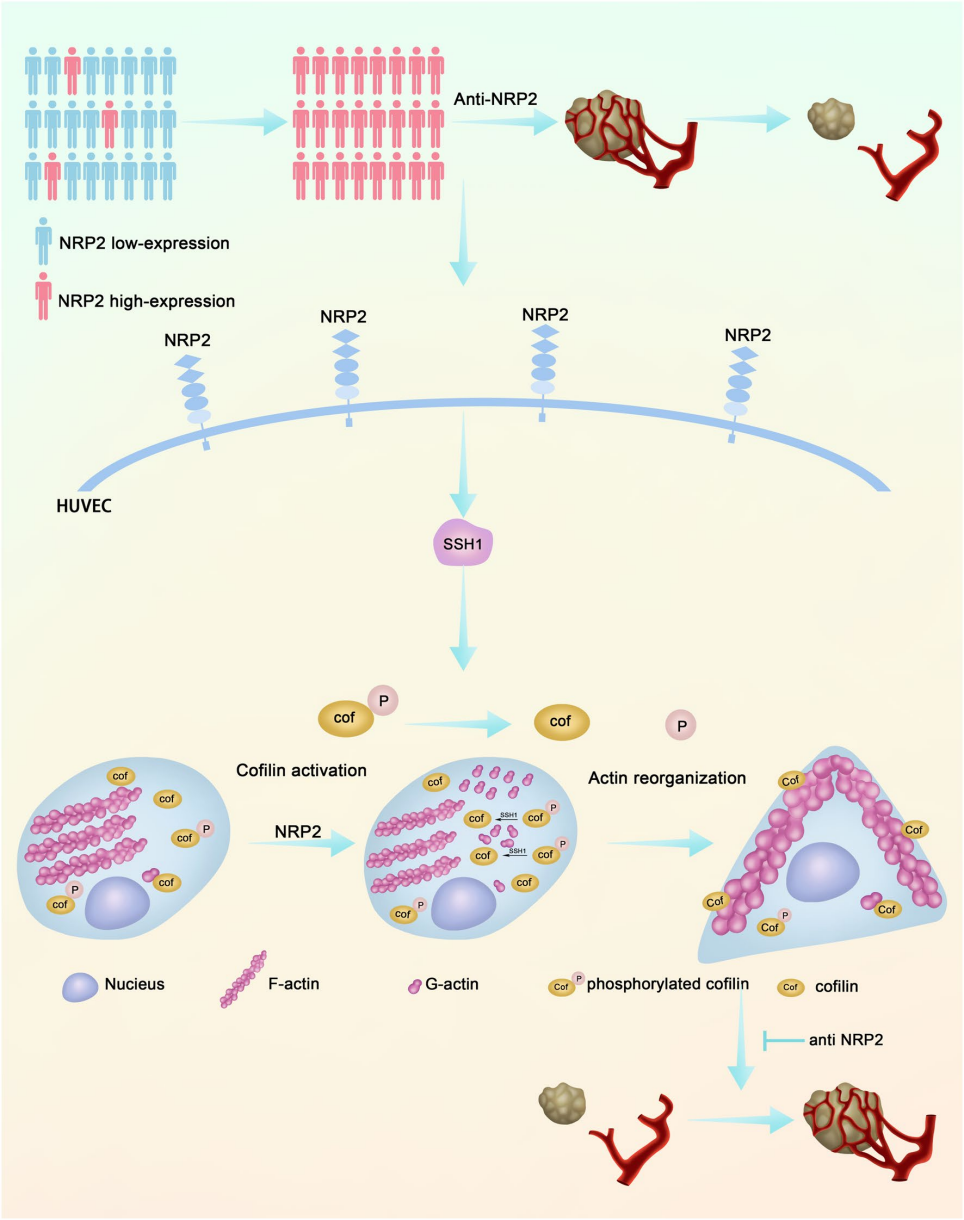
Proposed model of vascular NRP2 triggers PNET angiogenesis via activating SSH1-cofilin pathway

Angiogenesis is essential for tumor growth and is therefore a major step in PNET tumorigenesis [27]. Currently available clinical anti-angiogenic agents, which mainly target the VEGF/VEGFR2 pathway, are ineffective against PNETs [4]. Thus, there is a need to identify novel anti-angiogenic targets that could be used therapeutically. In this study, we demonstrated that NRP2 overexpression promoted the migration of HUVECs cultured in PNET conditioned medium. More importantly, we revealed a potentially significant role of NRP2 in PNET angiogenesis and tumor growth in an in vivo animal model (Fig. 6) and clinical specimens (Fig. 1). Overexpression of NRP2 regulated actin reorganization, leading to tube formation (Fig. 2, 3). These morphological changes were mediated by SSH1-enhanced cofilin dephosphorylation (Fig. 4, 5). Therefore, our study identifies a potential role of NRP2 in PNET angiogenesis and indicates that the NRP2/SSH1/cofilin axis is a potential target for preventing PNET progression(Fig. 7).

Cofilin is a known potent regulator of actin filament dynamics [28], and its ability to bind and polymerize actin is abolished by phosphorylation at Ser-3 [29]. For example, overexpression of LIMK1 in carcinoma cells significantly promotes cofilin phosphorylation, which then abolishes cancer cell motility to decrease invasion and metastasis in breast cancer [30]. By contrast, we showed that NRP2 upregulated cofilin activity via dephosphorylation at Ser-3 (Fig. 4, 5), resulting in actin polymerization and recruitment to the leading edge (Fig. 3e). Additionally, we observed that NRP2 enriched cofilin in the detergent-insoluble fraction (Fig. 3G(a)). Cofilin regulation and its effects on actin polymerization are responsible for cell motility and metastasis [31]. Indeed, according to our data, NRP2 promoted cofilin activity in PNET-associated HUVECs, which resulted in increased actin polymerization, membrane protrusion, and cell migration. In PNETs, the role of NRP2 in cofilin-mediated actin organization has not been reported previously. Our findings revealed a novel mechanism whereby NRP2 induced PNET angiogenesis via dephosphorylation of cofilin at Ser-3, suggesting that NRP2 antagonism may prevent angiogenesis and tumor growth in PNETs.

Cofilin dephosphorylation can be achieved by a variety of phosphatases, with CIN and SSH1 being the most important ones. Regarding transcription, an RNA array dataset showed that SSH1 amplification was positively correlated with NRP2 amplification (Fig. 5a). Moreover, SSH1 expression and transcription were increased by NRP2-induced cofilin dephosphorylation (Fig. 5b, c and Additional file 1. Figure S1b). Thus, NRP2 may regulate SSH1 transcription to increase its expression. A previous study showed that NRP2, a transmembrane protein, can dissociate from the membrane to the cytoplasm in tongue cancer and colon cancer cells [32, 33]. In eukaryotic cells, macromolecules such as proteins and RNA in the cytoplasm are separated from the translational machinery in the nucleus by nuclear pore complexes [34]. Therefore, we propose that NRP2 translocates into the nucleus and increases SSH1 transcription in PNET-associated vascular endothelial cells.

In conclusion, our evidence demonstrates that NRP2 is a promising selective target for undermining PNET angiogenesis and vascular integrity. Blocking NRP2 provides a potential clinical strategy in advanced PNET patients for overcoming the limitations of current vascular therapies that target VEGF/VEGFR2. It is urgent that we develop NRP2-specific inhibitors and evaluate them in clinical trials to increase the survival of PNET patients.

## Conclusions

PNET is robustly resistant to current anti-angiogenic therapies; thus, the angiogenesis mechanism of PNET urgently needs to be clarified. We found that NRP2 positively correlated with PNET vascularity and promoted F-actin polymerization by activating SSH1-cofilin, which resulted in HUVEC migration. Blocking NRP2 in vivo suppressed PNET angiogenesis and tumor growth, and elevated NRP2 expression was associated with poor prognosis in PNET patients. Our findings demonstrate NRP2 to be an important regulator of angiogenesis and a potential therapeutic target of anti-angiogenesis therapy for PNET.

## Methods

### Patients and specimens

A total of 13 PNET specimens, 7 non-small-cell lung cancer specimens and 7 colon cancer specimens were collected for analysis. Specimens were fixed in formalin and embedded in paraffin at the Diagnostic Histopathology Laboratory of Southwest Hospital of Third Military Medical University. All patients consented to an institutional review board-approved protocol that allows for the comprehensive analysis of tumor samples (Ethics committee of Southwest Hospital, Third Military Medical University (Army Medical University), Chongqing). This study conforms to the Declaration of Helsinki.

### Mouse model

Four-week-old female nu/nu mice were approved for use by the Institutional Animal Care and Use Committee of the Third Military Medical University (Chongqing, China). Animal care was provided in accordance with the Guidelines for the Care and Use of Laboratory Animals. To establish xenografts, 3 × 10^7^ BON cells in 100 µL of PBS and 100 µL of Matrigel were intraperitoneally injected into nude mice. After the tumor size reached ~ 100 mm^3^, the animals were assigned randomly to 2 groups (control group, PBS; and treatment group, anti-NRP2 antibody), with 2 mice per group. The body weights of the mice were similar in each group on the assignment day. The treatment group received intraperitoneal injection of 50 µg anti-NRP2 antibody (R&D AF567) every other day 4 times (total amount, 200 µg); control mice received equivalent injections of PBS. Tumor sizes were observed every other day. The mice were sacrificed 7 weeks after BON cell injection, and the xenografts were harvested and placed in 10% formalin for section preparation. The xenograft volume was calculated as V_T_ = [ l (length) × w^2^ (width)] × 0.52. Nude mice without tumors were excluded.

### Cell lines and culture conditions

BON cells were grown in DMEM/F12 medium supplemented with 20 mM L-glutamine and 10% foetal bovine serum. βTC3 cells were cultured in RPMI 1640 medium supplemented with 2 mM L-glutamine and 10% foetal bovine serum (100 µg/mL). HUVECs and HUVECs overexpressing NRP2 were grown in F-12 K medium supplemented with 10% foetal bovine serum. All cell lines were cultured in medium supplemented with 100 µg/mL penicillin and 50 µg/mL streptomycin at 37 °C under an atmosphere of 5% CO_2_ and passaged using standard cell culture techniques.

### Western blot analysis

Western blotting was performed with antibodies targeting the following proteins: VEGF receptor 2 (2479S, Cell Signaling), NRP2 (ab185710, Abcam), phospho-VEGF receptor 2 (Tyr951) (4991S, Cell Signaling), CD31 (ab28364, Abcam), CD34 (ab81289, Abcam), GAPDH (5174S, Cell Signaling), cofilin (5175S, Cell Signaling), phosphorylated cofilin (Ser3) (3313S, Cell Signaling), and SSH1 (ab76943, Abcam). Cells were lysed in RIPA buffer containing a protease inhibitor mixture (Roche) and incubated on a rocker at 4 °C for 15 min. The protein concentration of the lysates was measured using a BCA protein assay kit (Qiagen), and equal amounts of protein were separated by SDS-PAGE through 10% gels, transferred to PVDF membranes and probed with the indicated primary antibodies. Then, the blots were incubated with species-specific HRP-conjugated secondary antibodies, and the immunoreactive bands were visualized by enhanced chemiluminescence (ECL, Pierce). Three independent experiments were performed.

### Cellular F-actin/G-actin assay

F-actin and G-actin fractions were obtained using an F-actin/G-actin assay kit (BK 037, Cytoskeleton). Cells were scraped in LAS2 buffer containing detergents to disrupt the cell membrane before they were gently homogenized to lyse the cells. Then, the lysates was centrifuged at 350 × *g* (approx. 2000 rpm in a table-top microfuge) for 5 min at room temperature to pellet unbroken cells and tissue debris. Next, 100 µL of the resulting supernatant was centrifuged at 100,000 × *g* for 1 h at 37 °C to separate F-actin from soluble G-actin. Finally, the supernatant and pellet were analysed for actin content (G-actin in the supernatant versus F-actin in the pellet) by Western blot.

### Phalloidin staining

Whole-cell phalloidin staining was performed according to the manufacturer's protocol (Sigma P5282). Nuclei were stained with 4′,6-diamidino-2-phenylindole (DAPI) and viewed on an Olympus IX71 microscope.

### Analysis of Triton-soluble and insoluble actin

To measure Triton-soluble actin, cytoskeletal proteins were extracted and subjected to Western blot analysis with the indicated antibodies as previously described [20].

### Immunohistochemistry

Paraffin-embedded tissue sections were routinely dewaxed, rehydrated, and prepared for immunohistochemistry. Antigen retrieval was performed using sodium citrate, after which the sections were incubated in H_2_O_2_ (3%) for 10 min to block endogenous peroxidase activity. Next, the sections were blocked in 1% bovine serum albumin for 60 min, incubated with primary antibody at 4 °C overnight, and incubated with corresponding secondary antibody for 60 min. The specimens were treated with H_2_O_2_-diaminobenzidine until the desired staining intensity was observed before they were counterstained with haematoxylin, dehydrated and mounted. The results were verified by 2 independent individuals. Immunohistochemical staining was evaluated in accordance with the immunoreactive score (IRS), in which IRS = staining intensity (SI) X percentage of positive cells (PP). The SI was scored as follows: negative SI = 0; weak SI = 1; moderate SI = 2; and strong SI = 3. Similarly, the percentage of PP was scored as follows: > 10% PP = 0; 10% PP = 1; 11–50% PP = 2; 51–80% PP = 3; and > 80% PP = 4. An IRS ≥ 6 was identified as “high” expression, and an IRS < 6 was identified as “low” expression. Immunohistochemistry was performed with an antibody against CD31 (ab28364, Abcam), a rabbit monoclonal antibody against VEGF receptor 2 (2479S, Cell Signaling), and a rabbit polyclonal antibody against NRP2 (ab185710, Abcam).

### Plasmid construction, retrovirus infection, and stable cell line establishment

The human full-length open reading frame of NRP2 mRNA (NM_003872) was synthesized and integrated into the *AgeI/EcoRI* site of the pGC-LV-GV308 plasmid. For packaging, the lentiviral expression plasmid was cotransfected into HEK293T cells along with the helper plasmids pHelper 1.0 and 2.0. Culture media containing viral particles were harvested 48–72 h later and treated with polybrene before infection of HUVECs. Cells stably transduced with the lentiviral expression vectors were selected by culture in the presence of 2 µg/mL puromycin for 2 weeks. Stably transduced cell lines were seeded in 6-well plates at 140,000 cells per well. To induce the expression of the NRP2 transgene, cells were treated with 12 µg/mL doxycycline for 2 days. A stable cell line transfected with an empty vector was established as a negative control.

### siRNA transfection

siRNAs against NRP2, cofilin, and SSH1 and a non-targeting siRNA (siCtrl) were purchased from Shanghai Genechem Co., Ltd. All transfection experiments were performed using Lipofectamine RNAi MAX (Invitrogen) according to the manufacturer’s instructions. Briefly, HUVECs expressing the pGC-LV-GV308 plasmid containing empty vector or NRP2 (in 6-well plates) were treated with 12 µg/mL dox and conditioned medium from BON or βTC3 cells and were cultured to 70–80% confluence. Then, the cells were incubated with siRNA duplexes against NRP2, cofilin or SSH1 in Opti-MEM (Invitrogen). The medium was replaced with fresh F-12 K medium 6 h after transfection. Cells were harvested 48 h after transfection to determine the mRNA and protein levels. The following siRNA sequences were used: SSH1, 5′ UCGUCACCCAAGAAAGAUA 3′; cofilin, 5′ AAGUCUUCAACGCCAGAGGAG 3′; NRP2, 5′-AAAGGCTGGAAGTCAGCACTAATTT-3′; and scrambled siRNA, 5′-AAAGGAGGGGCATGCCACGTTGG-3′.

### Apoptosis assay

HUVECs stably transfected with NRP2 were treated with the indicated medium for 24 h. Then, the cells were harvested, double stained with propidium iodide (PI) and FITC-Annexin V and further analysed by flow cytometry (BD Biosciences) to evaluate apoptosis rates.

### Wound-healing assay

For the wound-healing assays, HUVECs stably expressing empty vector or NRP2 were previously treated with 12 µg/mL dox, seeded into 6-well cell culture plates with complete medium and cultured to ~ 100% confluence. After 6 h of serum starvation, an artificial, homogenous wound was created by scratching the monolayer with a sterile 200-µL pipette tip. Images of cells migrating into the wound were captured under a microscope 24 h and 48 h after scratching.

### Capillary tube formation assay

For the tube formation assay, Matrigel (Corning, #354248) was dissolved at 4 °C overnight, added to each well of prechilled 96-well plates (100 µL/well) and incubated for 45 min at 37 °C. HUVECs expressing empty vector or NRP2 were previously treated with 12 µg/mL dox, resuspended in conditioned medium from BON or βTC3 cells, plated at a density of 1 × 10^4^/well and cultured for 12 h in a humidified 5% CO2 atmosphere. After 3.5, 4, and 6 h, the capillary-like structures of HUVECs were photographed under a light microscope, and the images were stored on a computer. Tubular structures were quantified by manual counting at 100 × magnification.

### Gene set enrichment analysis

Gene Set Enrichment Analysis (GSEA) software (https://software.broadinstitute.org/gsea/index.jsp) was used to determine whether a previously defined set of genes (from the GO, KEGG, and Reactome databases) showed significant differences in expression between the MLP and IT groups [21, 22]. A heatmap of 394 genes from GO: 0030029 was produced using R project (https://www.r-project.org/). The enlarged figure of the heatmap was drawn with genes exhibiting significant expression differences (fold change > 2, *P* < 0.001) between the MLP and IT groups.

### Statistical analysis

Statistical significance was determined by two-tailed Student’s *t* test. Error bars represent the SD, as indicated in each figure legend. All experiments were repeated at least three times (biological replicates) with consistent results; however, the figures show one representative experiment (with an average of the technical replicates). Statistical significance is indicated by asterisks in the figures, as follows: *P < 0.05; **P < 0.005; and ***P < 0.0005.

## Supplementary information

**Additional file 1:** **Figure S1.** **a** HUVECs were cultured in the presence or absence of conditioned medium from BON cells (treatment and control, respectively ) or in the presence of conditioned medium from beta-TC3 cells. Then, these cells were transfected with empty vector or an NRP2 overexpression plasmid before they were seeded for the capillary tube formation assay. Representative images at 3.5 h and 4 h and after plating are shown. **b** RNA was isolated and analysed by RT-PCR. The results were normalized to the levels in the empty vector group (n = 3, mean ± SEM).

**Additional file 2:** **Figure S2.** **a** After HUVECs were transfected with empty vector or an NRP2 overexpression plasmid, they were cultured in conditioned medium from BON cells, A549 cells or SW480 cells for 24 h, after which they were seeded in plates. A wound-healing assay was performed, and images were captured at 0 h and 48 h after scratching. **b** Statistics of the migration rate of HUVECs cultured in conditioned medium from BON, A549 or SW480 cells.

**Additional file 3:** **Figure S3.** **a** HUVECs were treated with conditioned medium from BON cells. Then HUVEC-scramble-siRNA or HUVEC-cofilin-siRNA cells were transfected with empty vector or an NRP2 overexpression plasmid. The cells were then subjected to a wound-healing assay. **b** Representative image of the wound-healing assay using HUVECs transfected with NRP2 either alone or with the cofilin S3E mutant. **c** Representative image of the wound healing assay using HUVECs transfected with NRP2 either alone or with the cofilin S3A mutant.

**Additional file 4:** **Figure S4.** **a** Xenograft mouse models of CRC and lung cancer were established with SW480 cells and A549 cells, respectively. After the mice were injected with anti-NRP2 antibody or PBS for the indicated schedule, the xenografts were dissected and assessed. **b** Tumor sizes were measured every other day after injection with PBS or NRP2 antibody.

## Data Availability

All data generated or analysed during this study are included in this published article (and its supplementary information files).

## Abbreviations

NRP2: Neuropilin 2
PNET: Pancreatic neuroendocrine tumor
SSH1: Cofilin phosphatase slingshot-1
SEMA3: Type 3 semaphorin
F-actin: Filamentous actin
LIMKs: LIM kinases,
TESKs: Testicular protein kinases
CIN: Haloacid dehalogenase termed chronophin
CRC: Colorectal cancer
NSCLC: Non-small-cell lung cancer
HUVECs: Human umbilical vein endothelial cells
