# Supplementary figures and images for: Vascular NRP2 triggers PNET angiogenesis by activating the SSH1-cofilin axis
Source: Cell Biosci. 2020 Sep 23;10:113. doi: 10.1186/s13578-020-00472-6 (PMC7509939; doi:10.1186/s13578-020-00472-6)

Supplemental figure 1

A

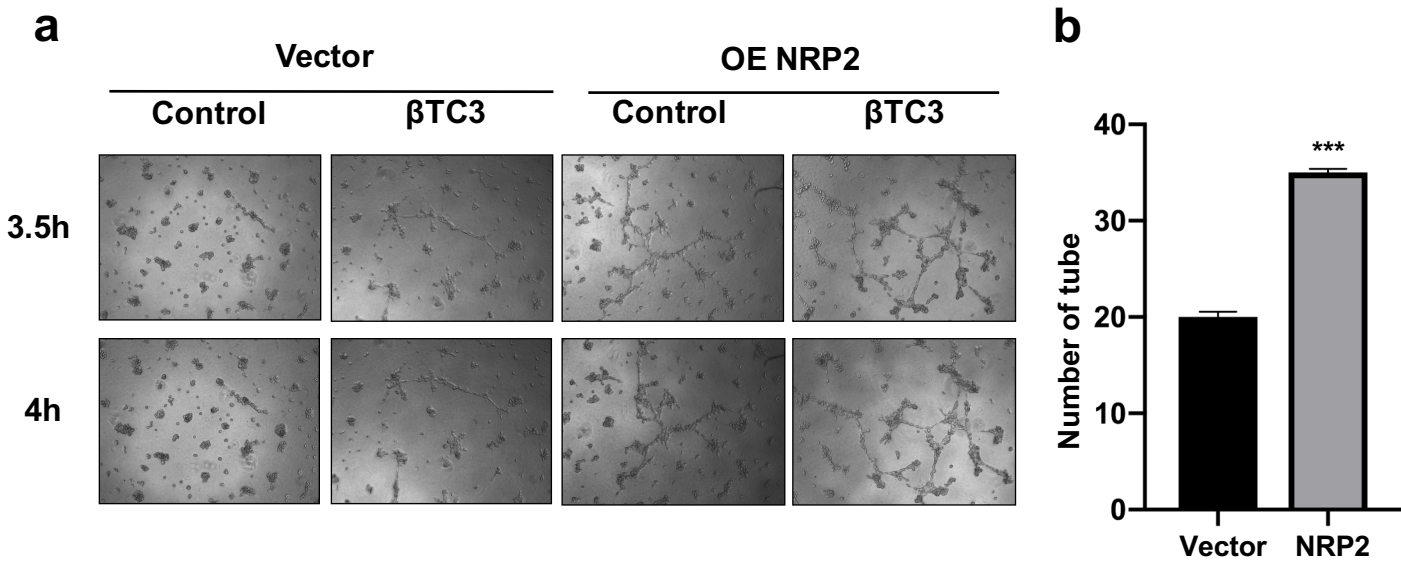

B

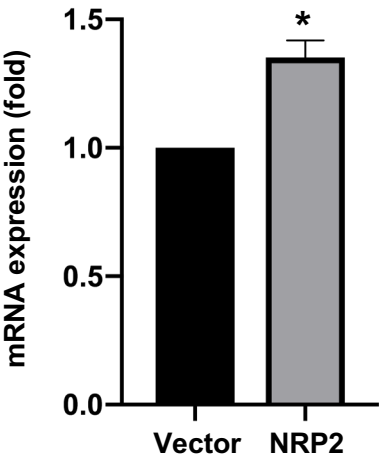

Supplement: Supplementary file 1 — Additional file 1: Figure S1. a HUVECs were cultured in the presence or absence of conditioned medium from BON cells (treatment and control, respectively ) or in the presence of conditioned medium from beta-TC3 cells. Then, these cells were transfected with empty vector or an NRP2 overexpression plasmid before they were seeded for the capillary tube formation assay. Representative images at 3.5 h and 4 h and after plating are shown. b RNA was isolated and analysed by RT-PCR. The results were normalized to the levels in the empty vector group (n = 3, mean ± SEM). [file 13578_2020_472_MOESM1_ESM.pdf]

Supplemental figure 2

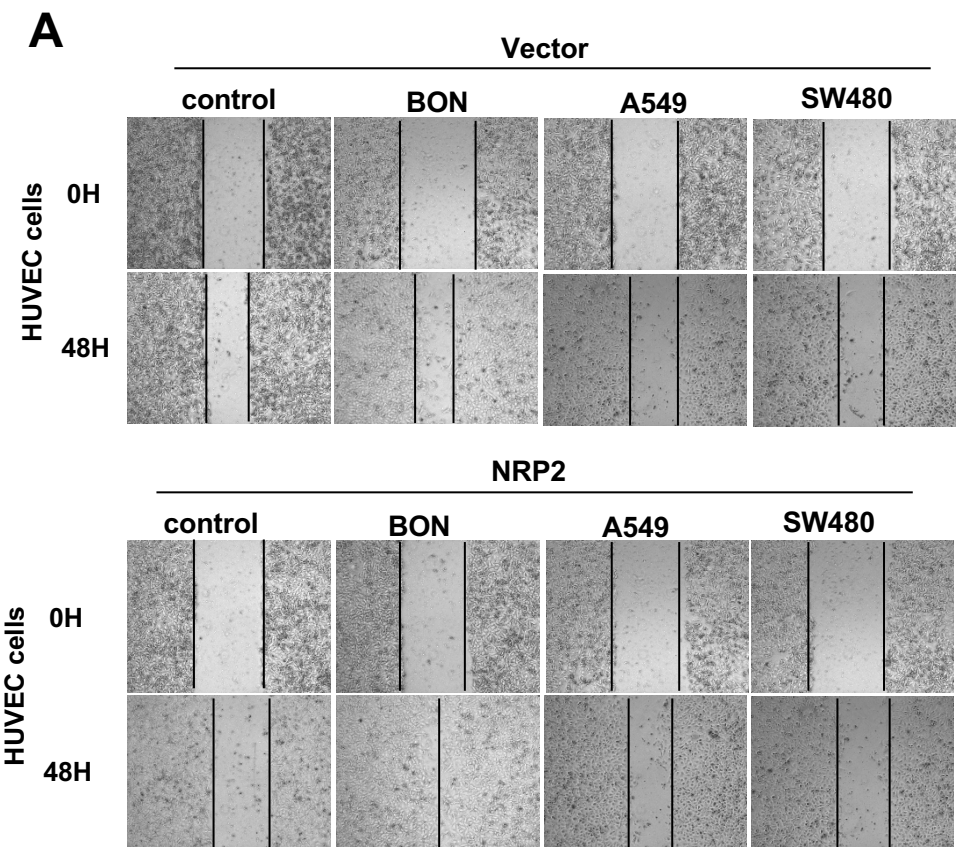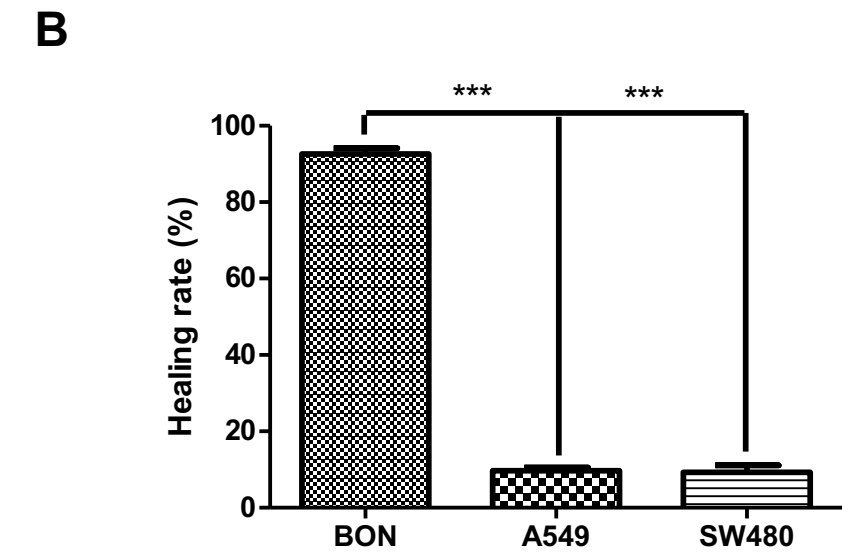

Supplement: Supplementary file 2 — Additional file 2: Figure S2. a After HUVECs were transfected with empty vector or an NRP2 overexpression plasmid, they were cultured in conditioned medium from BON cells, A549 cells or SW480 cells for 24 h, after which they were seeded in plates. A wound-healing assay was performed, and images were captured at 0 h and 48 h after scratching. b Statistics of the migration rate of HUVECs cultured in conditioned medium from BON, A549 or SW480 cells. [file 13578_2020_472_MOESM2_ESM.pdf]

Supplemental figure 3

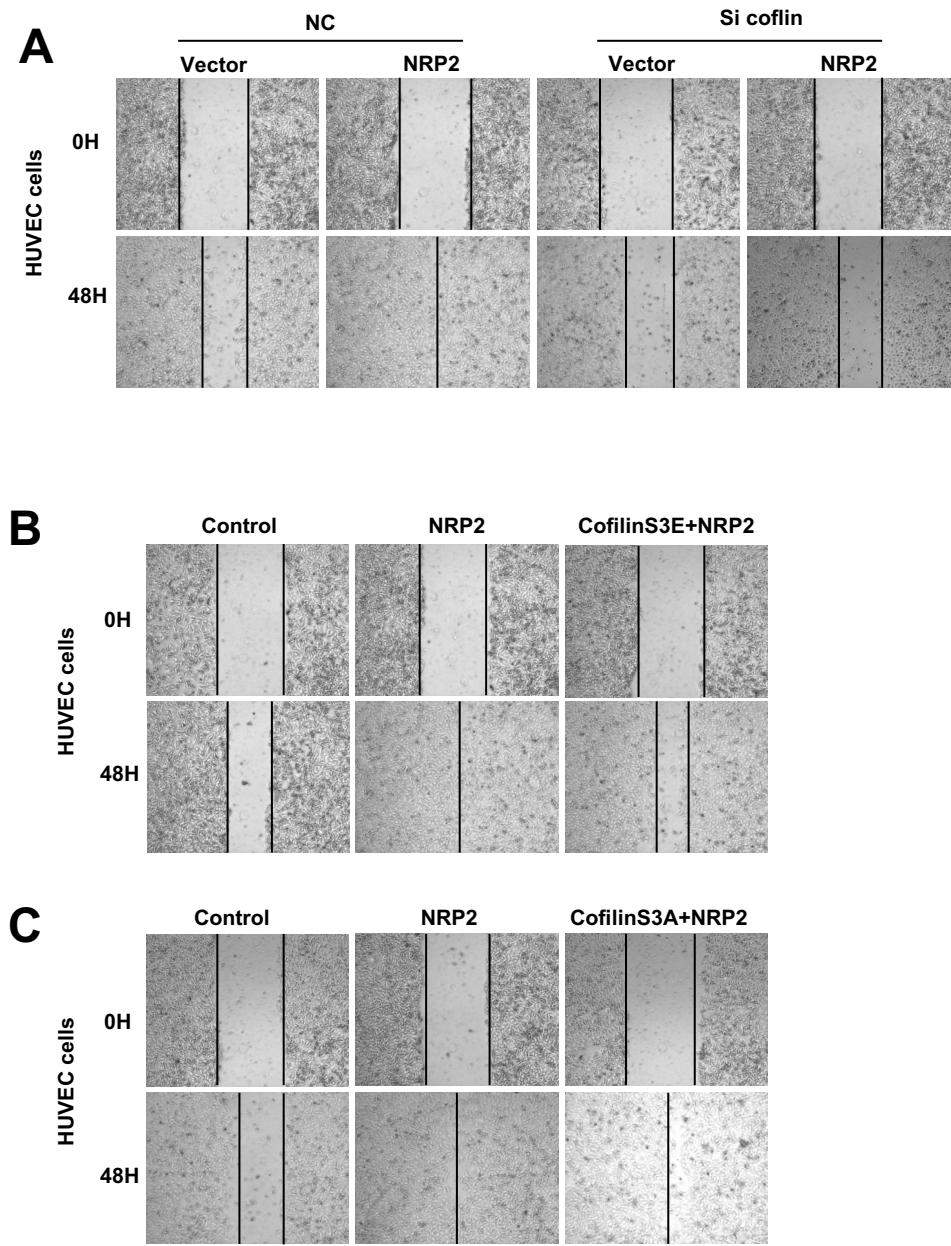

Supplement: Supplementary file 3 — Additional file 3: Figure S3. a HUVECs were treated with conditioned medium from BON cells. Then HUVEC-scramble-siRNA or HUVEC-cofilin-siRNA cells were transfected with empty vector or an NRP2 overexpression plasmid. The cells were then subjected to a wound-healing assay. b Representative image of the wound-healing assay using HUVECs transfected with NRP2 either alone or with the cofilin S3E mutant. c Representative image of the wound healing assay using HUVECs transfected with NRP2 either alone or with the cofilin S3A mutant. [file 13578_2020_472_MOESM3_ESM.pdf]

Supplemental figure 4

A

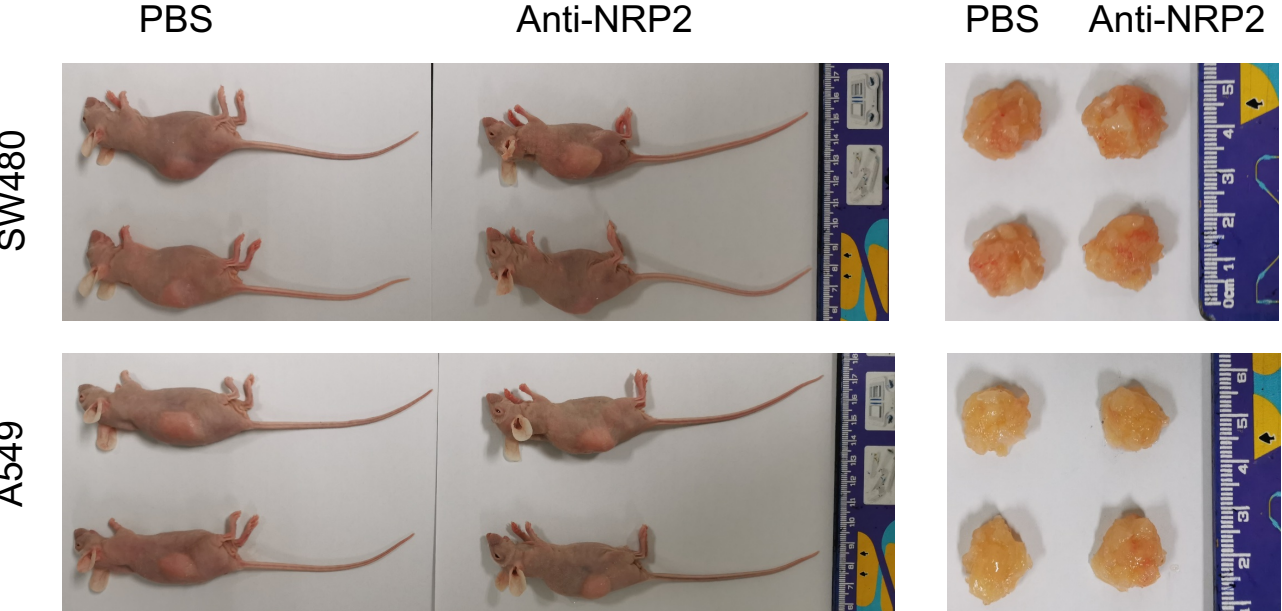

B

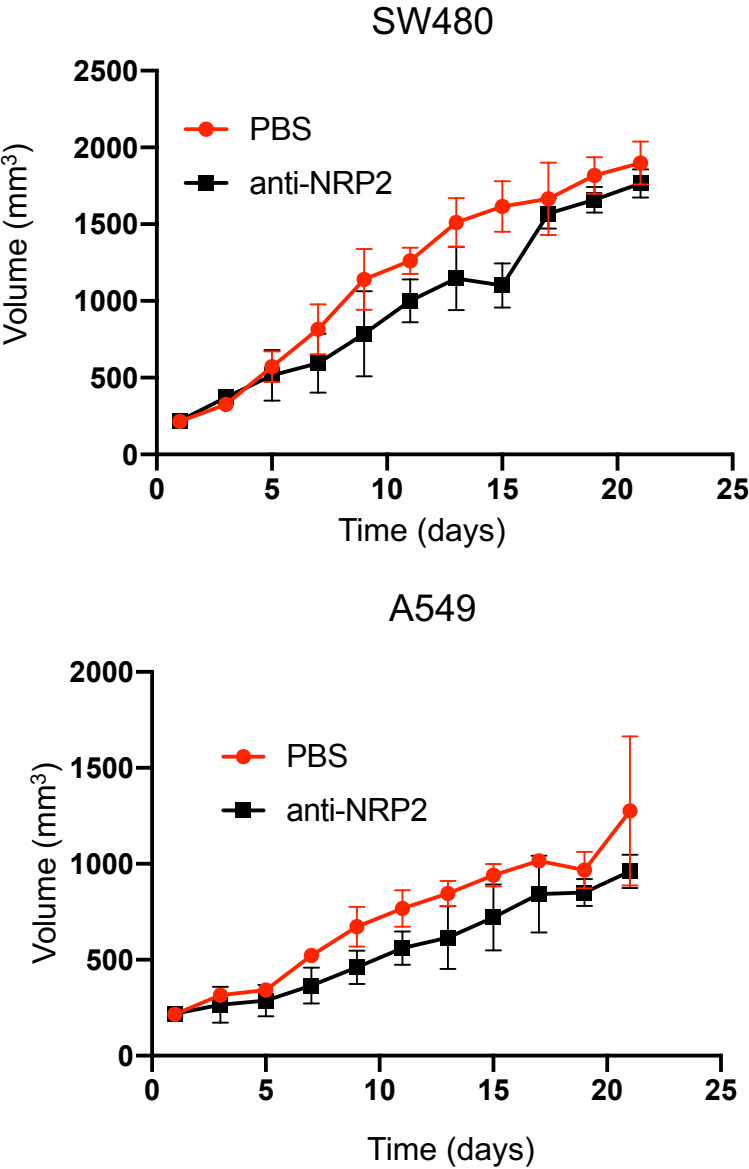

Supplement: Supplementary file 4 — Additional file 4: Figure S4. a Xenograft mouse models of CRC and lung cancer were established with SW480 cells and A549 cells, respectively. After the mice were injected with anti-NRP2 antibody or PBS for the indicated schedule, the xenografts were dissected and assessed. b Tumor sizes were measured every other day after injection with PBS or NRP2 antibody. [file 13578_2020_472_MOESM4_ESM.pdf]
